# “Your Friends Do Matter”: Peer Group Talk in Adolescence and Gender Violence Victimization

**DOI:** 10.3390/children8020065

**Published:** 2021-01-20

**Authors:** Sandra Racionero-Plaza, Elena Duque, Maria Padrós, Silvia Molina Roldán

**Affiliations:** 1 Department of Sociology, University of Barcelona, 08034 Barcelona, Spain; sandraracionero@ub.edu; 2 Department of Theory and History of Education, University of Barcelona, 08035 Barcelona, Spain; 3 Department of Teaching and Learning and Educational Organization, University of Barcelona, 08035 Barcelona, Spain; mariapadros@ub.edu; 4 Department of Pedagogy, Universitat Rovira i Virgili, 43007 Tarragona, Spain; silvia.molina@urv.cat

**Keywords:** adolescence, gender violence, coercive dominant discourse, socialization, peer group, healthy relationships

## Abstract

Research on gender violence has identified as one main component leading to gender violence a dominant socialization process which associates attractiveness to men who show violent behaviors and attitudes, while egalitarian and non-violent men are emptied of attractiveness. This is known as coercive dominant discourse. Starting from the evidence that the peer group is a main context of socialization in adolescence, quantitative data were collected from six classes of secondary education (14–15-year-old adolescents) to explore whether the coercive dominant discourse is displayed in social interactions in the peer group and, if so, how it influences attractiveness patterns and sexual-affective behavior in adolescence. The analyses reveal that the coercive dominant discourse is often reproduced in the peer group interactions, creating group pressure, and pushing some girls to violent relationships. Alternative ways of interaction are also reported, which allow a socialization leading to more freedom, less coercion, and more healthy relationships.

## 1. Introduction

Data on gender violence show that, in the many forms it can take, it is part of the life stories of many women from early ages. It can be found either in stable or sporadic relationships, from current or ex-partners. Gender violence is a structural problem in current societies that makes prevention and victim protection difficult when some of these forms of violence are perceived as normal and receive social tolerance [1]. Globally, the United Nations [2] showed that one in three women (35%) around the world had suffered physical and/or sexual violence by their partner or ex-partner or sexual violence by others at some point in their lives. In Europe, one in five women has been a victim of physical and/or sexual violence from her current partner or ex-partner, one in 10 women has been a victim of sexual violence (including girls under age 15), and one in 20 women has been raped. Regarding psychological violence, the 43% of women declared to have suffered some form of this violence from their partner or ex-partner [3]. In the context where this research was conducted, there is a similar situation. According to data from the Spanish Ministry of Health, Social Services and Equality [4], adolescents and youth between 16 and 24 years old have suffered physical violence from their partner or ex-partner (10.3%), being severe violence in half of the cases, sexual violence (6.8%), psychological violence of control from their current partner (19.4%) or from a former partner (41.9%), emotional psychological violence with ex-partners (29%) or with their current partner (7.4%), and economic violence (7.3%). Importantly, data from 2017 showed that the highest increase in gender violence as compared to the previous year was in the age group of women under the age of 18 (14.8% of increase, while the overall increase rate was 2.6%) [5].

Because of the concern about the high number of victims of gender violence and their decreasing age [6], research from various perspectives has delved in the cause of this problem. From an evolutionary point of view, research [7] has found that aggressive behavior and certain physical traits benefit men’s sex partner accumulation; for example, enhanced masculine facial characteristics can increase both perceived dominance and negative attributions, with those aspects resulting in being successful for men [7]. Social and socio-cultural perspectives are complementary to evolutionary approaches. Research in the social area has found a dominant model of socialization that links sexual attractiveness to dominant models of masculinity that have violent attitudes and behaviors [8,9]. This socialization is produced through mass media, TV series, movies, youth literature, social media, etc., which tend to present as sexually attractive a male model with sexist, racist and non-democratic values and behavior, who despises, humiliates and mistreat others [10,11], while egalitarian non-violent men and boys are emptied of attractiveness [12]. This entails a coercive dominant discourse [13] that can affect youths’ sexual-affective preferences and choices in the immediate and long term. Research has shown that this coercive dominant discourse is reproduced among female adolescents, as they prefer boys with violent attitudes and behaviors, mostly for sporadic relationships and non-violent boys for stable relationships [14]. Studies have also evidenced that the coercive dominant discourse can be transformed when cognitive tools for the critical analysis of this discourse are provided [15].

Studies in various social sciences have shown an increase in the attraction towards violent young males [16], which highlights the vulnerability of young women that engage in relationships with those men. It has been found that some girls feel attracted by aggressive people, even when they recognize that these people are violent [16,17]. In this regard, hooks [18] explained that some people feel attached to those who mistreat, and when the relationship turns destructive, it is difficult for them to leave it, even tolerating behaviors that they would not tolerate in friendship. However, research also shows that it is precisely this violent nature of the relationship which breeds desire: when the ‘good boy’ is defined by girls as attractive, fun, romantic, confident, etc., he is seen as ‘too good’ and therefore less attractive by some girls [19]. In consistence with this evidence, research in the field of criminology shows that being engaged in delinquency raises the number of dates, increasing the attractiveness of delinquents [20]. 

All of this research relates to the importance of male role models in adolescent sexual-affective socialization and gender violence victimization. In this regard, three different types of masculinity have been identified, with a different role in the perpetuation or overcoming of gender violence [12]: the dominant traditional masculinity (DTM), the oppressed traditional masculinity (OTM) and the new alternative masculinities (NAM). The first type (DTM) includes those men characterized by domination and inequality; although not all DTM are violent, all men who are violent with women are DTM. Neither OTM nor NAM are violent. OTM men are seen as the “good boys” who are not considered attractive because of a lack of self-confidence, and therefore they are not an alternative to DTM to overcome gender violence. On the contrary, NAM are those non-violent men who show themselves as self-confident, who actively position themselves against gender violence, and therefore are seen as attractive and constitute an alternative to overcome gender violence. Associating attractiveness to the egalitarian attitudes of the new alternative masculinities is a key component of the prevention of attraction to violent attitudes [21]; on the contrary, a coercive dominant discourse that places attractiveness on dominant traditional masculinities and disinterest and boredom on oppressed traditional masculinities contributes to perpetuate gender violence; on the one hand, by promoting girls’ attraction towards dominant boys and, on the other, by presenting these boys as male role models that can put pressure on boys to follow this model to enhance their attractiveness and their sexual-affective success.

Sexual-affective socialization is especially important during adolescence and preadolescence, when this topic becomes a major concern for boys and girls, and they have their first sexual-affective relationships, and because the first learning about love has the most impact in subsequent relationships [22]. While acknowledging multiple agents of influence on adolescents’ social and emotional development, such as home environment, research in developmental psychology has indicated that the peer group is the main context of socialization in adolescence, and interactions in the peer group, what adolescents talk about and how they talk in relation to sexual-affective relationships, are key components of the sexual-affective socialization process of adolescents, as such communicative interactions contribute to shaping expectations on gender issues [23,24]. For instance, research has found that jealousy, one main cause of conflict in teen couples, is socially normalized among adolescents, as a normal and necessary expression of love, and this contributes to learning that violent behavior is compatible with love, and even proof of it [25]. In addition, some studies [26,27] highlighted the pressure exerted in some cases by the peer group of girls on a girl to start a relationship with a boy. In the case of boys, the peer group can also have a key role, for instance, in promoting girls being despised [28]. Research has also shown that social networks can have a key role in spreading the aggressive behaviors of participants in these social interactions [29]. Therefore, the influence of peer interactions has a crucial role for learning attraction to violence or attraction to non-violence and equality, and in the subsequent choice of violent or non-violent partners for stable or sporadic relationships.

In the ground of prior results from the research line on preventive socialization of gender violence regarding socialization into the dominant coercive discourse that associates attraction to violent and aggressive males, and considering the role of the peer group in adolescents’ socialization, the study reported in this manuscript is novel in exploring whether the coercive dominant discourse is displayed in communicative interactions in the adolescent peer group and, if so, how it influences attractiveness patterns and sexual-affective behavior among adolescents.

## 2. Materials and Methods

### 2.1. Research Design

This study was conducted in the framework of the broader project MEMO4LOVE: Social interactions and dialogues that transform memories and promote sexual-affective relationships free of violence from high schools [30]. More specifically, the study reported here was part of the project’s objective 2: to analyze the characteristics of communicative interactions between adolescents in the context of secondary education schools that mediate the learning of attraction to violence, and the characteristics of those communicative interactions that mediate the learning of attraction to equality and wellbeing.

The object of study includes psychological and social processes that take place spontaneously in natural environments and cannot be observed while they occur without causing interference. In addition, we are analyzing not only the types of interactions that occur among adolescents, but also how they influence boys’ and girls’ thoughts and preferences, which entails a subjective dimension of the phenomenon. For this reason, data collection strategies focused on gathering adolescents’ perceptions and interpretations of the interactions that occur in the peer group and the effects that these have. 

For this purpose, a series of variables to analyze the coercive dominant discourse were established by the researchers informed by the state of the art on this topic, which were used to create the data collection instrument that was developed to specifically address the aforementioned research objective. These variables included: changes in adolescents’ attraction and types of peer group interactions related to such changes; peer group pressure to engage in a sexual-affective relationship, type of relationship started as a result of such pressure, type of arguments used in peer group pressure and characteristics of the boys with whom the relationship started after pressure; type of masculinity preferred and group talk about attractive/convenient people. Data on these variables were collected based on adolescents’ responses, which were subsequently analyzed and interpreted by the researchers to identify associations between these variables that could indicate the existence or not of a coercive dominant discourse in the peer group interactions and, if existing, to define its main features. 

### 2.2. Sample

The sample was composed of 141 adolescents from six classes of secondary education in three different secondary schools in a southern city in Spain (two public schools and one private school). Overall, 60% of the participants were girls and 40% were boys. Moreover, 93% of the adolescents in the sample were aged 14 (50%) or 15 (43%), although some students were already 16 (4%) or 17 (1%). 

Previous to adolescents’ participation in the study, the selected schools were informed about the research objectives and potential social impact of the research, as well as the participation terms. Once the schools accepted to participate, the selected classrooms were informed about the project, and then informed consent from the participant adolescents’ parents or legal tutors, and informed assent from the participant adolescents were obtained. Anonymity and confidentiality were ensured throughout the data collection and analysis processes, as well as the psychological wellbeing of the participants. For this purpose, the research followed article 8 of the Charter of Fundamental Rights of the European Union about personal data protection, and the Directive 95/46/EC regarding the fundamental rights and freedom of persons and their right to privacy in personal data protection. The study was fully approved by the Ethics Committee of Andalusia Government (Secure verification code: eb4d0ea33a6f286ed96b583f094459bb1cb9678e).

### 2.3. Data Collection Instrument

Data were collected through a questionnaire designed in the framework of the MEMO4LOVE project. The questionnaire focused on the type of people that the peer group consider attractive, how the peer group talk about these people, and how peer interactions have an impact on adolescents’ preferences and choices in their first sexual-affective relationships. The questionnaire was composed of a total of 44 questions structured into 9 sections: 0. Personal data; 1. Ideas about attraction and love; 2. Characteristics of the people and relationships that are considered attractive or convenient; 3. Models of masculinity; 4. Mirage of upward mobility; 5. Peer group pressure; 6. Changes in attraction; 7. Characteristics of the boys you like; 8. Open question. These sections were created taking as references the main theoretical contributions on the preventive socialization of gender violence [8,12,21,31]. Questions were close-ended with multiple choice answers to allow quantitative analyses, except for the final open question, which allowed participants to add other relevant information. The questionnaire aimed at analyzing the characteristics of the communicative interactions among adolescents in relation to attraction to violence or to non-violence; therefore, most of the questions refer to “what the peer group thinks” or “how peers talk in the group”, and only in some of the questions (sections 6 and 7 mentioned above) participants are asked about their personal opinion. To facilitate the data collection, the questionnaire was responded to individually in the adolescents’ classroom during school hours.

To ensure content-related validity, the questionnaire underwent a validity expert judgement by experts in preventive socialization of gender violence—these experts were members of the Advisory Council of the project—and subsequently, the instrument was piloted with a small sample of adolescents of the same age as the study sample. As a result, the writing of some questions was refined, and the final version of the questionnaire was obtained. 

For the purpose of analyzing the coercive dominant discourse in the peer group, 13 of the questions were analyzed, about the following topics: Group talk about attractive/convenient people, Group pressure to engage in a relationship, Influence of peer group interactions on adolescents’ attraction, and Preference for a type of masculinity. Responses about the Influence of peer group interactions on adolescents’ attraction and about the Preference for a type of masculinity referred to respondents’ own experience and preferences, while responses about the other topics referred to talk and interactions in the peer group.

### 2.4. Data Analysis

Data were analyzed with the SPSS statistical package. The responses obtained from the selected questions were analyzed using descriptive statistics, first each question individually and then crossed with the responses of other questions that could indicate manifestation of the coercive dominant discourse. Crosstabs were obtained, and frequencies and percentages were analyzed. 

To analyze some of the multiple-choice questions, the responses were grouped into categories to allow a more significant interpretation of the data in terms of the objective of the study. This is the case of questions asking for characteristics (adjectives) describing group talk about certain boys and girls. Those adjectives were grouped into: adjectives reflecting violence (aggressive, possessive, tough, cocky, dominant, bad), adjectives reflecting non-violence (respectful, egalitarian, caring, sensitive, good), and neutral adjectives in terms of violence (romantic, confident, attractive, funny, flirt, strong, sweet, insecure, others). For the case of those questions, percentages were based on the responses and not on the cases (respondents).

## 3. Results

### 3.1. Discourse in the Peer Group Influences Adolescents’ Preferences

More than half of the adolescents surveyed stated that the way that they talk with their friends has sometimes made them see other people differently and change the attractiveness that they perceive in these people. Overall, 58% of the adolescents perceived this influence. In the cases when this influence of the peer group interactions was experienced, it occurred in the two opposite directions, with similar probability: to favor start or stop liking one particular person. When thinking of the last time that this happened, 49% of adolescents stated that it was a person that they did not like or did not attract them and started liking or feeling attracted to them as a consequence of the group influence, while 51% of adolescents stated that it was a person that they liked or considered attractive and started disliking or perceiving as not attractive as a consequence of the group influence. 

When asked about the causes of this change in adolescents’ preferences and attraction, according to their perceptions, the main cause they identified is the way in which the peer group talked about that person, either highlighting positive attributes and values (39% of the respondents) or negative attributes and values (37% of the respondents). When the peer group talked about that person as attractive or interesting (24% of the respondents) or as not attractive or interesting (13% of the respondents), it also influenced adolescents’ preferences, although less importantly. It is relevant to highlight that the main reasons are related to verbal interactions in the group—how the peers talk about that person—while other type of interactions, such as the behavior that the peer group shows with that person—showing their interest (18% of the respondents) or disinterest (11% of the respondents) on what that person did—were less relevant. 

When the type of change in adolescents’ preference—start liking or start disliking—is analyzed in relation with the cause identified for this change, we can see that the direction of the change in adolescents’ preference coincides with the type of group talk about that person. When they started to like a person, 68% of adolescents stated that it was because the peer group highlighted qualities that they perceived as positive, 44% stated it was because of the peer group talked about that person as being attractive or interesting, and 27% stated that it was because the peer group showed interest on what that person said or did. When they stopped liking a person, 69% of adolescents stated that it was because the peer group highlighted qualities that they perceived as negative, 21% stated that it was because the peer group talked about that person as not being attractive or interesting, and 17% stated that it was because the peer group did not show interest on what that person said or did. (see Table 1).

These data suggest that it is mainly via verbal interactions and discourse in the peer group that the group influences adolescents’ preferences.

#### The Preference for a Type of Masculinity is Related to Peer Group Talk about Attractive and Convenient People for a Relationship

Peer group talk also influences preferences for certain types of masculinity. This is important because different types of masculinity have a different role in the perpetuation or elimination of gender violence (OTM, DTM, NAM) [12]. Most of the adolescents who are attracted by boys (heterosexual girls or homosexual boys) said to prefer the NAM model for any type of relationship. In addition, although the informed preference for the DTM model was low, it was higher for sporadic (8%) than for stable (2%) relationships (see Table 2).

Importantly, the type of masculinity preferred for a partner in a relationship (sporadic or stable) seems to be related to the way that the peer group talks about the people who they consider attractive and the people who they think are convenient for a relationship. When the model of masculinity adolescents like is the DTM, peer group talk to describe the people that they perceive as attractive more frequently uses the adjectives associated with violence or the neutral adjectives than those associated with non-violence. This occurs similarly when DTM is the preferred model for a stable relationship or for a sporadic relationship, as the percentage of adjectives related to non-violence (21% for stable and 23% for sporadic relationships), violence (37% for stable and 32% for sporadic relationships) and neutral (42% for stable and 45% for sporadic relationships) are similar in both cases. When the masculinity model adolescents like is OTM, peer group talk to describe the people who the group perceive as attractive more frequently uses adjectives related to non-violence or neutral adjectives than those related to violence. Again, the pattern is similar when OTM is the preferred model for a stable relationship (28% non-violence, 16% violence, 56% neutral adjectives) or for a sporadic relationship (37% non-violent, 13% violent, 50% neutral adjectives). Finally, when the masculinity model adolescents like is NAM, non-violence adjectives and neutral adjectives are also the most frequently used by the peer group to describe attractive people, being the pattern similar when adolescents prefer NAM for a stable (41% non-violence, 14% violence, 44% neutral) or for a sporadic relationship (42% non-violent, 14% violent, 44% neutral). We can see that the higher use of non-violence adjectives when talking about attractive people in the peer group occurs when adolescents prefer NAM boys, while the highest use of violence adjectives occurs when adolescents prefer DTM boys. These data indicate that those adolescents that prefer DTM boys for a relationship are more frequently involved in peer talk that associates attractive people to violence, while those who prefer OTM and especially those who prefer NAM boys are less involved in peer talk that associates attractiveness with violence, and more in talk that associates attractiveness to non-violence (see Table 2).

Regarding group talk about convenient people for a relationship, the adjectives related to violence appear more frequently when adolescents prefer DTM boys, but the percentages are much lower than occurred regarding group talk about attractive people (13% when they prefer DTM for a stable relationship and 16% when they prefer DTM for a sporadic relationship). For adolescents who prefer OTM or NAM boys, the use of violence adjectives ranges only between 0% and 3%. Non-violence adjectives are more frequently used when adolescents prefer NAM (59% when adolescents prefer NAM for a stable relationship and 62% when they prefer NAM for a sporadic relationship), although there is little difference with the use of these adjectives when adolescents prefer DTM (50% when they prefer them for a stable relationship and 50% for a sporadic one) or OTM (54% when they prefer them for a stable relationship and 53% for a sporadic one). The neutral adjectives are more frequently used when adolescents prefer OTM (46% when they prefer them for a stable relationship and 44% for a sporadic one). These data indicate that, when talking about convenient people in the peer group, adjectives related to violence are much less present than when talking about attractive people, and adolescents who prefer DTM boys are the ones mainly exposed to such discourse (see Table 2).

These data suggest that, for the sample studied, peer group talk does reproduce the coercive dominant discourse. On the one hand, because the peer group talk is different when talking about attractive or convenient people in a relationship, reproducing the double standard that persists in society that there are, on the one hand, “good” persons that are convenient but people do not feel attracted by them and, on the other hand, “attractive” persons who are desired, but are not good or convenient. On the other hand, peer group talk reproduces the coercive dominant discourse because adolescents that prefer boys that represent a model of masculinity characterized by violence and disrespect are those most involved in peer group talk that present violent people as people that can be chosen for a relationship, reproducing the coercive dominant discourse in society that violent people—and particularly violent men—are desirable partners for a relationship. 

### 3.2. The Coercive Dominant Discourse in the Peer Group Influences Adolescents’ Sexual-Affective Relationships

The peer group also has an influence on adolescents’ sexual-affective choices and decisions and, in this domain, we also identify components of the coercive dominant discourse. Adolescents were asked if they knew cases of boys or girls having started a relationship as a result of peer group pressure. Specifically, the questions referred to pressure by female friends in the case of girls and by male friends in the case of boys, so we obtained data regarding group pressure by same sex friends to engage in sexual-affective relationships.

#### 3.2.1. The Type of Relationship Started as a Result of Group Pressure is Related to the Way (More or Less Violent) that the Peer Group Perceives the Boy/Girl

Data show that there is peer group pressure to start a sexual-affective relationship. Both boys and girls are pressured to start a relationship with another person. Nonetheless, according to adolescents’ experiences, girls are more often pressured by the group to start a relationship with a boy: 55% of participants (*n* = 77) are aware of at least one case of a girl being pressured to have a relationship with a boy, while 47% (*n* = 66) of participants are aware of at least one case of a boy being pressured to have a relationship with a girl. These data show the higher vulnerability of girls to peer group coercion for engaging in sexual-affective relationships.

In addition, when a relationship starts as a consequence of group pressure, in most cases, the type of relationship initiated was sporadic, non-stable, both in the case of boys (in the 86% of adolescents who identified group pressure to a boy leading to the onset of a relationship it was a sporadic one) and girls (69% of known cases of girls starting a relationship as a result of group pressure started a sporadic relationship). 

Data also allow analyzing the characteristics of the boys—as perceived by the participants’ peer group—with whom girls started a relationship because of group pressure. The adjectives selected by the adolescents to describe those boys were different depending on the type of relationship initiated: sporadic or stable. When girls start a stable relationship with a boy as a result of group pressure, most of the adjectives associated with the perception of the group of that boy are adjectives describing non-violence (40% of all adjectives selected) or neutral (43%); a smaller percentage (17%) of adjectives are related to violence. However, when girls start a sporadic relationship as a consequence of group pressure, the neutral adjectives in terms of violence to describe the boy are the most frequently selected (55%), and the use of adjectives related to violence rises (21%). That is, the peer group tend to push girls to engage in stable relationships with boys that the group do not tend to perceive as violent, but push girls to engage in sporadic relationships with boys that the group see more frequently as violent (see Table 3).

These data seem to indicate a relationship between the way that the peer group perceives the boy with whom the group pressures a girl to engage in a sexual-affective relationship, and the type of relationship that the girl eventually ends up having. This may be because adolescents would associate different types of persons with different types of relationships. The higher use of adjectives related to violence in the cases when girls start a sporadic relationship as a result of group pressure highlights the higher vulnerability of girls to violence in these relationships. Moreover, this is important because, as mentioned, most of the relationships started as a result of group pressure are sporadic relationships. The association between the characteristics perceived in boys and the type of relationship that the girls start with them can be related with a coercive dominant discourse in the peer group that may be reproducing the double standard that persists in society. Yet, more importantly, the peer group would be exerting a coercive discourse that would compromise girls’ wellbeing and healthy relationships by pushing them to sporadic relationships with boys with violent behaviors and attitudes.

#### 3.2.2. The Type of Argument Used for Peer Group Pressure is Related to the Way (More or Less Violent) the Peer Group Perceives the Boy

The type of argument used by the peer group to pressure girls to engage in a relationship also helps in describing the type of coercive discourse used. When girls have a relationship as a result of group pressure, the most frequent argument used by her friends was that he was a good boy (48% of the respondents), followed by the argument to have the experience (45%), that he was a popular boy (29%) and that he is a boy with experience with other girls (29%). Looking at the adjectives more frequently chosen to describe the boy according to the group perspective, adjectives related to non-violence were more frequently selected when the argument was that he was a good boy (40% of the adjectives) than for the other arguments: 23% for the argument to have the experience, 18% for the argument that he was a boy with experience with other girls, and 16% for the argument that he was a popular boy. Conversely, adjectives related to violence were more frequently chosen when the arguments were that he was a popular boy (25% of the adjectives), that he was a boy with experience with other girls (29%), or for the argument to have the experience (28%), than for the argument he was a good boy (10%) (see Table 4).

Besides the logical association between recommending someone because he is a good boy and describing him as non-violent, we identify high frequent use of arguments related with an instrumental conception of relationships (being popular or having experience). In the case of girls engaging in a relationship because of group pressure, these arguments have a low occurrence only when the boy is described with non-violent adjectives, which suggests that engaging in a sexual-affective relationship with a boy perceived as non-violent may protect them from superficial and instrumental relationships, that is, from the harmful effect of coercive dominant discourse in the peer group.

## 4. Discussion

The data analyzed show that a coercive dominant discourse is displayed in the social interactions in the peer groups within the sample of adolescents involved in this study. This discourse took different forms, including influence in adolescents’ sexual-affective preferences, in their attractiveness towards certain types of masculinity, in adolescents’ sexual-affective behaviors and choices via peer group pressure to start a relationship, and in reproducing the double standard that exists in society that tends to associate certain characteristics of people to certain types of relationships. All these forms of the coercive dominant discourse contribute to adolescents’ socialization in the attraction towards violent male models, increasing the risk of gender violence victimization and compromising adolescents’ healthy affective and sexual relationships.

Our data showed that non-violent caring boys were associated with stable relationships, while aggressive dominant boys were associated with sporadic relationships. This is consistent with previous research that found that boys with violent attitudes and behaviors are preferred for sporadic relationships, while non-violent boys are preferred for stable relationships, which is an important risk for gender violence victimization in sporadic relationships [14]. The present study adds the important role that social interactions in the peer group have to create this vulnerability through generating situations of peer group pressure and coercion. Our data reported group pressure of same-sex friends, that is, group pressure on girls being exerted by other girls. Previous research has already pointed to the reality of girls starting a relationship with a boy as a result of female peer group pressure [26,27], but our research has expanded such knowledge by indicating that the content of such pressure from female friends differs when pressuring for stable or sporadic relationships. The pressure for a sporadic relationship occurred most often with a violent boy, while the pressure for a stable relationship was most of the time with a “good boy”. As mentioned, this raises the risk for gender violence victimization in sporadic relationships. At the same time, in line with other research [14], it dismantles the idea that stable relationships, often associated with the idea of romantic love, are the problem and essential source for gender violence victimization. Our findings indicate that stable relationships can be more often relational contexts that protect from gender violence victimization. 

The type of arguments used in peer group pressure to start a relationship showed a frequent instrumentalization of those adolescents with whom the relationship was started, using the reason that they were popular, had experience, or that the relationship could be used to gain experience. Research has demonstrated that quality relationships contribute to a better health, while instrumental relationships based on popularity, money or power damage health [32,33]. Data on neuroscience and adolescents also show that adolescents with lower quality friendships and a reduced support network tend to engage in a higher number of risk behaviors, while adolescents with more close friendships and support tend to engage in a lower number of risk behaviors [34]. Therefore, our results are relevant and have implications, not only from the perspective of the social and psychological development of adolescents and their education, but also from a point of view of public health. 

Although there is a percentage of responses that report the argument of being a “good boy” for group pressure on girls to start a sporadic relationship, these responses have to be put in the context of previous research [14], which has shown that boys with violent attitudes and behaviors, and not “good” boys, are preferred for sporadic relationships. In this regard, further research including qualitative data could clarify what “good boys” meant for adolescents in that context and how it relates to the association between sporadic relationships and violence. Moreover, one can interpret that result as social desirability in the respondent adolescents, i.e., you must choose what is ethically acceptable, which is non-violence. Ethically, you cannot say I told my friend to have a sexual relationship with a boy because he was violent. Third, the result can also be interpreted as a retelling of a different story to oneself (naming “good” a violent boy) as a way to justify pressure on others to have a sporadic relationship with violent boys or for oneself to have a relationship with such type of boys. Prior research [35] can also sustain this interpretation. 

Interactions in the peer group seemed to influence more attraction and desire, but not the idea of the type of persons that are convenient, showing that the “language of desire” is more effective at influencing adolescents’ sexual-affective preferences and choices than the “language of ethics” [36]. This is not a negative result, but a key finding to be considered in educational and health preventive programs aimed at preventing gender-based violence in adolescence. To be more effective, those programs should employ more language of desire than language of ethics, or at least a combination of both but not only language of ethics, that is, the language of what is “good” and “bad” in the sexual-affective realm. If the peer group uses language of desire to talk about who is liked and why, professionals should speak the same language, while employing scientific evidence, to enter the dialogue with adolescents. 

Our results confirm the existence of coercive dominant discourse in the peer group in the sample of adolescents surveyed, showing a worrying picture that contributes to the reproduction of gender violence among adolescents from the group of ‘friends’ via communicative interactions. However, our findings also provide a positive note: peer group discourse can be also an element of transformation. Previous studies found that choosing transformative interactions helps in identifying violence situations, placing value on support and friendship, and promoting the helpful role of allies [37,38]. Research has already demonstrated that one factor related to the occurrence of sexual violence against children and adolescence in the school context is the lack of educational interventions on this topic [39]. In this regard, educative and health interventions with adolescents that promote a critical analysis of the coercive dominant discourse can have a transformative effect [15]. Our findings highlight the role that peer group dialog has in this transformation, which has clear implications for educational programs. According to evidence, in interventions to prevent gender violence, it is crucial to draw on peer group social interactions to transmit children and adolescents the values, desires, feelings and friendship relationships that are likely to promote the choice for relationships free of violence, and thus a freer and healthier life. Consequently, in terms of implications, preventive educational and health programs should intervene at the peer group level to tackle the networks of social interactions and development that are most relevant in adolescents’ sexual socialization. 

Study limitations are related, on the one hand, to the fact that the data and analysis performed do not allow one to establish a causal relationship between the variables explored. Subsequent research could contribute with additional data and analyses that confirm causal relationships between peer group talk and the coercive dominant discourse that contribute to gender violence in adolescence. Moreover, qualitative research could complement this quantitative analysis to deepen adolescents’ meanings and perceptions regarding how the coercive dominant discourse operates in their peer groups. On the other hand, the sample is relatively small, and geographically concentrated. Subsequent research could be conducted with a broader sample that allows generalizing the conclusions.

## Figures and Tables

**Table 1:** Changes in adolescents’ sexual-affective preferences and peer group discourse.

		Change in Adolescents’ Attraction for a Boy or a Girl as a Result of Peer Group Interaction					
		(a) Started Liking Him/Her		(b) Stopped Liking Him/Her		Total	
		F	%	F	%	F	%
Reasons for adolescents’ change in attraction (up to 3 options could be selected)	(a) The peer group highlighted positive qualities and values in him/her	28	68%	4	10%	32	39%
	(b) The peer group highlighted negative qualities and values in him/her	2	5%	29	69%	31	37%
	(c) The peer group said that he/she was attractive or interesting	18	44%	2	5%	20	24%
	(d) The peer group said that he/she was not attractive or interesting	2	5%	9	21%	11	13%
	(e) The peer group was interested in what that person did or said	11	27%	4	10%	15	18%
	(f) The peer group was not interested in what that person did or said	2	5%	7	17%	9	11%
	(g) Don’t know /Not sure	7	17%	9	21%	16	19%
	Total respondents	41	49%	42	51%	83	100%

**Table 2:** Type of masculinity preferred for stable or sporadic relationships and peer group talk about attractive and convenient people.

		Type of Boy Chosen as the Most Attractive to Have a Stable Relationship (Responded Only by Adolescents Who Like Boys)						Type of Boy Chosen as the Most Attractive to Have a Sporadic Relationship (Responded Only by Adolescents Who Like Boys)					
		(a) DTM		(b) OTM		(c) NAM		(a) DTM		(b) OTM		(c) NAM	
		F	%	F	%	F	%	F	%	F	%	F	%
Total respondents		2	2%	9	11%	74	87%	7	8%	17	20%	59	71%
		F	%	F	%	F	%	F	%	F	%	F	%
Adjectives used in the peer group to talk about attractive people (up to 5 options could be selected):	Non- violence adjectives	4	21%	12	28%	142	41%	7	23%	28	37%	118	42%
	Violence adjectives	7	37%	7	16%	50	14%	10	32%	10	13%	39	14%
	Neutral adjectives	8	42%	24	56%	153	44%	14	45%	38	50%	125	44%
	Total adjectives	19	100%	43	100%	345	100%	31	100%	76	100%	282	100%
Adjectives used in the peer group to talk about convenient or appropriate people to have a relationship with (up to 5 options could be selected):	Non- violence adjectives	8	50%	22	54%	194	59%	16	50%	41	53%	162	62%
	Violence adjectives	2	13%	0	0%	10	3%	5	16%	2	3%	2	1%
	Neutral adjectives	6	38%	19	46%	124	38%	11	34%	34	44%	97	37%
	Total adjectives	16	100%	41	100%	328	100%	32	100%	77	100%	261	100%

**Table 3:** Type of relationship started by a girl as a result of group pressure and peer group perception of the boy.

		Type of Relationship Started by a Girl as a Result of Group Pressure			
		(a) A Sporadic Relationship		(b) A Stable Relationship	
		F	%	F	%
Adjectives describing the peer group’s perception of the boy with whom a girl started a relationship as a result of peer group pressure (up to 5 options could be selected)	Non-violence adjectives	52	23%	44	40%
	Violence adjectives	48	21%	18	17%
	Neutral adjectives	124	55%	47	43%
	Total adjectives	224	100%	109	100%

**Table 4:** Type of argument used for peer group pressure on girls and peer group perception of the boy.

		Arguments Used by Friends to Pressure Girls to Start a Relationship with a Boy							
		(a) He Is a Good Boy		(b) He Is a Popular Boy		(c) He Is a Boy with Experience with Other Girls		(d) To Have the Experience	
		F	%	F	%	F	%	F	%
Total respondents		37	48%	22	29%	22	29%	35	45%
		F	%	F	%	F	%	F	%
Adjectives describing the peer group’s perception of the boy with whom a girl started a relationship because of peer group pressure (up to 5 options could be selected)	Non-violence adjectives	72	40%	15	16%	18	18%	36	23%
	Violence adjectives	18	10%	24	25%	29	29%	44	28%
	Neutral adjectives	90	50%	57	59%	53	53%	77	49%
	Total adjectives	180	100%	96	100%	100	100%	157	100%

## Data Availability

The data presented in this study are available on request from the corresponding author.
